# 3D Analysis of Ordered Porous Polymeric Particles using Complementary Electron Microscopy Methods

**DOI:** 10.1038/s41598-019-50338-2

**Published:** 2019-09-27

**Authors:** Juan Alvarez, Giovanni Saudino, Valentina Musteata, Poornima Madhavan, Alessandro Genovese, Ali Reza Behzad, Rachid Sougrat, Cristiana Boi, Klaus-Viktor Peinemann, Suzana P. Nunes

**Affiliations:** 1 King Abdullah University of Science and Technology (KAUST), Biological and Environmental Science and Engineering Division (BESE), Advanced Membranes and Porous Materials Center, 23955-6900 Thuwal, Saudi Arabia; 2 Alma Mater Studiorum-Università di Bologna, via Terracini 28, 40131 Bologna, Italy; 3 King Abdullah University of Science and Technology (KAUST), Core Labs, 23955-6900 Thuwal, Saudi Arabia; 4 King Abdullah University of Science and Technology (KAUST), Physical Science and Engineering Division (PSE), Advanced Membranes and Porous Materials Center, 23955-6900 Thuwal, Saudi Arabia

**Keywords:** Imaging techniques, Microscopy

## Abstract

Highly porous particles with internal triply periodic minimal surfaces were investigated for sorption of proteins. The visualization of the complex ordered morphology requires complementary advanced methods of electron microscopy for 3D imaging, instead of a simple 2D projection: transmission electron microscopy (TEM) tomography, slice-and-view focused ion beam (FIB) and serial block face (SBF) scanning electron microscopy (SEM). The capability of each method of 3D image reconstruction was demonstrated and their potential of application to other synthetic polymeric systems was discussed. TEM has high resolution for details even smaller than 1 nm, but the imaged volume is relatively restricted (2.5 μm)^3^. The samples are pre-sliced in an ultramicrotome. FIB and SBF are coupled to a SEM. The sample sectioning is done *in situ*, respectively by an ion beam or an ultramicrotome, SBF, a method so far mostly applied only to biological systems, was particularly highly informative to reproduce the ordered morphology of block copolymer particles with 32–54 nm nanopores and sampling volume (20 μm)^3^.

## Introduction

Porous particles are relevant for chromatography, sorption-selective protein separations, catalysis, sensors and controlled drug delivery. They are generated by different methods, which involve nucleation induced by a non-solvent, emulsification^1,2^, suspension^3^ and multistage polymerization, extractable porogens^4^ and the selective etching of assembled block copolymers, as reviewed by Gokmen and Du Prez^5^. The particles’ size can be controlled by the polymerization, by the surfactant compositions in emulsions, or by using microfluidic^6^ devices. A crosslinking can additionally strengthen the particle stability^7^. The total surface area and the full pore accessibility are essential for most applications.

The particles pore geometry and distribution are in most case random, but especially interesting is the particle formation with a highly ordered morphology, which can be facilitated by a block copolymer self-assembly^8,9^. The rich morphology of block copolymer systems is extensively investigated and exploited for different purposes, other than particles formation. We have been using block copolymers for membrane formation with narrow pore size distribution^10–12^ and tuned functionalization, and for the generation of biomimetic tridimensional structures^13,14^; block copolymers have been used as precursors, templates and base for the preparation of hybrid materials for energy conversion and storage^15^. The morphology is guided by the thermodynamic interactions between blocks, the block-solvent affinities and by the block fractions. Spheres, cylinders and lamellae are the simplest equilibrium morphologies observed in solvent-free diblock copolymer systems^16^. The structure complexity rapidly increases when a third or multi-blocks are added^9^. An even broader variety of morphologies can be seen when the copolymers are dispersed^17,18^ in or exposed to selective solvents. When quenched by the addition of non-solvents or a fast temperature change, non-equilibrium morphologies can be trapped. Nevertheless, the adopted morphology can be influenced by a confinement^19–21^ in thin layers, cylinders or in small particles, giving rise to frustrated phase-separated structures.

If kept in a metastable condition for a sufficient time, the systems can evolve, trapping non-equilibrium morphologies of low energy. An interesting case is that of triply periodic bicontinuous structures^22^, which were first reported for lyotropic liquid crystals surfactant-water systems^23^ and later for annealed solid block copolymer systems^8^. They result from the concurrent minimization of the contact area between incompatible copolymer chain segments and the maximization of the macromolecules conformation entropy. A perfect gyroid triply periodic minimal surface would be entirely composed by triple junctions with no straight lines. The observation and mathematical representation of minimal surfaces in surfactant systems have been proposed already by Plateau^24^. Schwarz theoretically described cubic symmetries like the primitive P and diamond D. Much later Schoen^25^ identified the gyroid G and I-WP symmetries. P, D and G are adjoint morphologies, with G and D being the most stable. In a system in evolution, it is possible that the structures coexist and convert to the most stable geometry as time proceeds. The formation of bicontinuous cubic structures in liquids has been described by Scriven by the interplay of intermolecular forces leading to a minimum^23^.

We prepared highly porous particles with remarkable order, by carefully inducing the phase separation of diluted solutions of linear polystyrene-*b*-poly (acrylic acid) copolymers with the addition of a poor solvent^26^. Later La *et al*.^27^ reported porous particles with cubosome structures, using branched-linear poly(ethylene glycol)-*b*-polystyrene copolymers. The two cases reflect interconnected geometries with constant mean curvatures. Bicontinuous structures are appealing for transport, sorption and catalytic applications^28^. Regular electron microscopy provides only a simplified and restricted image of complex 3D structures. The advent of high-resolution 3D image reconstruction methods can be extremely useful for these cases. We applied and compared for the first time a series of advanced and complementary methods of 3D imaging of highly ordered nanoporous particles with triply periodic minimal surfaces: transmission electron microscopy (TEM) tomography, slice-and-view focused ion-beam (FIB) and serial block face (SBF) scanning electron microscopy (SEM).

## Results and Discussion

### Particles formation and mechanism

**Figure 1:**
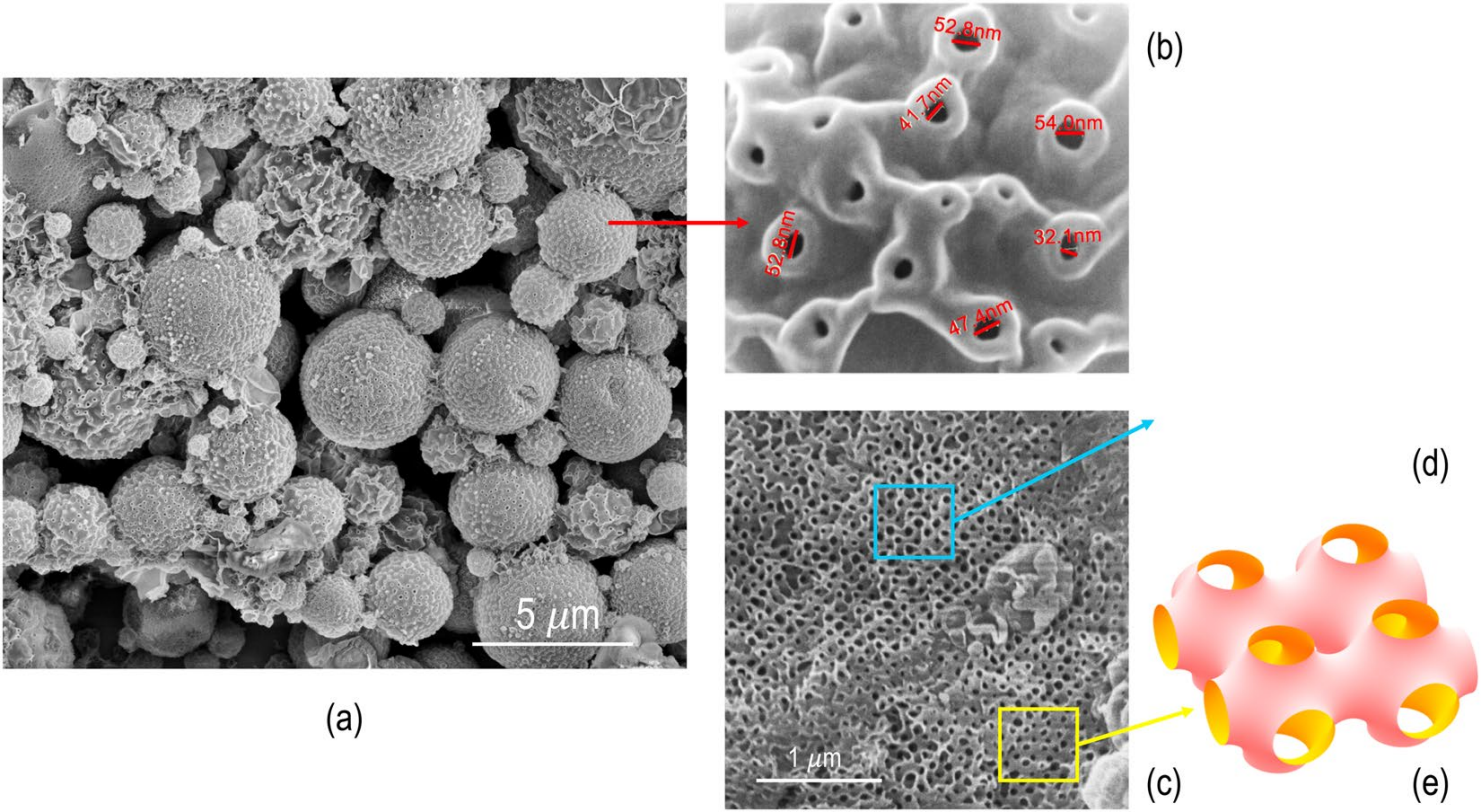
PS-*b*-PAA porous particles SEM images: (**a**) particles with different stages of evolution; (**b**) detail with high magnification of particle surface pores; (**c**) particle fracture, depicting areas with (**d**) Schoen gyroid and (**e**) Schwarz P morphology.

PS_15k_-*b*-PAA_1.6k_ porous particles were prepared by dispersing the polymer in a toluene/methanol mixture, followed by the addition of a larger amount of the poor solvent, methanol. The particles morphology is shown in Fig. 1a. Optimization tests were performed to scale-up the number of particles prepared in a single step. For the preparation of 10 mg of copolymer particles we initially used 1.2 mL of a 1:3 toluene/methanol solution. We observed that, to double the amount of prepared particles, when the amount of solvents and polymer were equally doubled, it resulted in an aggregation of the small non-porous particles. However, by keeping the same solvent quantity and ratios, and increasing the concentration of the polymer in solution, and the reaction time, a larger number of porous particles was obtained. In opposite to previous preliminary experiments^26^ conducted in small scale, we observed that 8 hours stirring were not enough to complete the particles formation. After this time lamellar and vesicle structures predominate. Therefore, we concluded that by increasing the amount of polymer a longer time around 15 hours was needed to conclude the particles formation with a stabilized pore structure. Most particles in Fig. 1a are completely formed, while part is still in the late stages of formation. The pore sizes on the particle surfaces are in the range of 32 to 54 nm, as seen in Fig. 1b with high magnification. The scale-up to a more concentrated dispersion reduced the time needed for the freeze-drying, centrifugation and sonicating procedures, and enabled the use of the same batch of particles to run different experiments. Figure 1c shows the internal image, facilitated by a particle fracture. A periodic ordered morphology is seen, which resemble in part a Schoen gyroid (Fig. 1d) or a Schwarz P structure (Fig. 1e).

Methanol is a poor solvent for PS_15k_-*b*-PAA_1.6k_. In the toluene/methanol mixture the system is kept in a metastable condition. Partial solubilization is promoted. A copolymer diluted solution coexists with nuclei of a copolymer concentrated phase. A slow process of self-assembly in the confined nuclei proceeds with the formation of triply periodic minimal surface morphologies, analogous to those observed in lipids and microemulsions. The ordered structures resemble primitive P cubic and gyroid lattices^27^.

### 3D Image characterization

The high-resolution microscopy images shown in Fig. 1 represent only part of the complex morphology of the obtained particles. Figure 1c is a strong indication of the presence of triply periodic minimal surface structures. However, a complete morphological representation can be only fulfilled with a 3D image reconstruction. We have previously demonstrated the advantages of 3D image reconstruction by slice-and-view FIB/SEM^29,30^ and SBF/SEM^30^ to fully characterize the morphology of polymeric membranes, obtaining information on their asymmetric porosity, as well as to reveal details of hierarchical porous isotropic films^13,14^. We apply the two methods and TEM tomography for the porous particles characterization for the full 3D image reconstruction in different scales and confirmation of the morphology.

**Figure 2:**
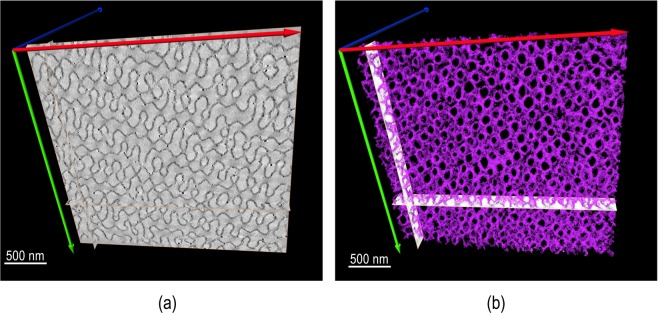
PS-*b*-PAA porous particles (**a**) TEM image and (**b**) and TEM tomography reconstruction of a 100 nm slice.

The TEM image of a thin slice of a porous particle embedded in epoxy resin and stained with RuO_4_ is shown in Fig. 2a. A periodic structure is seen. However, the regular pore morphology can be much better represented by the tomography in Fig. 2b.

The TEM tomography gives a clear representation of the pore size and distribution at high magnification, highlighting the center of one of the particles. However, for a complete overview of the porous structure in different particles other methods of 3D image are needed.

X-ray tomography is a non-destructive method of 3D image and reconstruction. An attractive advantage is the possibility of performing different *in situ* time resolved experiments, while imaging. It has been used to visualize pharmaceutical granules (>1 mm)^31^, monolithic porous columns with pores smaller than 1 μm^32^ combined with fluid dynamics computer simulations, water distribution in fuel cell systems^33^. The main disadvantage of x-ray tomography has been however that the resolution has been, so far, in the micrometer range and insufficient for nanostructured materials. This is being improved in new generations of lab equipment and synchrotron facilities. X-ray tomography with 0.4 μm resolution has been applied to image electrospun nanofibers of nylon and poly(L-lactic acids)^34^. Polymer composites have been imaged with resolution below 1 μm^35^. The characterization of LiFePO4 nanoplates for batteries with 10 nanometer resolution enabled by soft X-ray and of catalysts with 23 nm space resolution, using synchrotron-based ptychographic X-ray computed tomography have been recently reported^36,37^.

**Figure 3:**
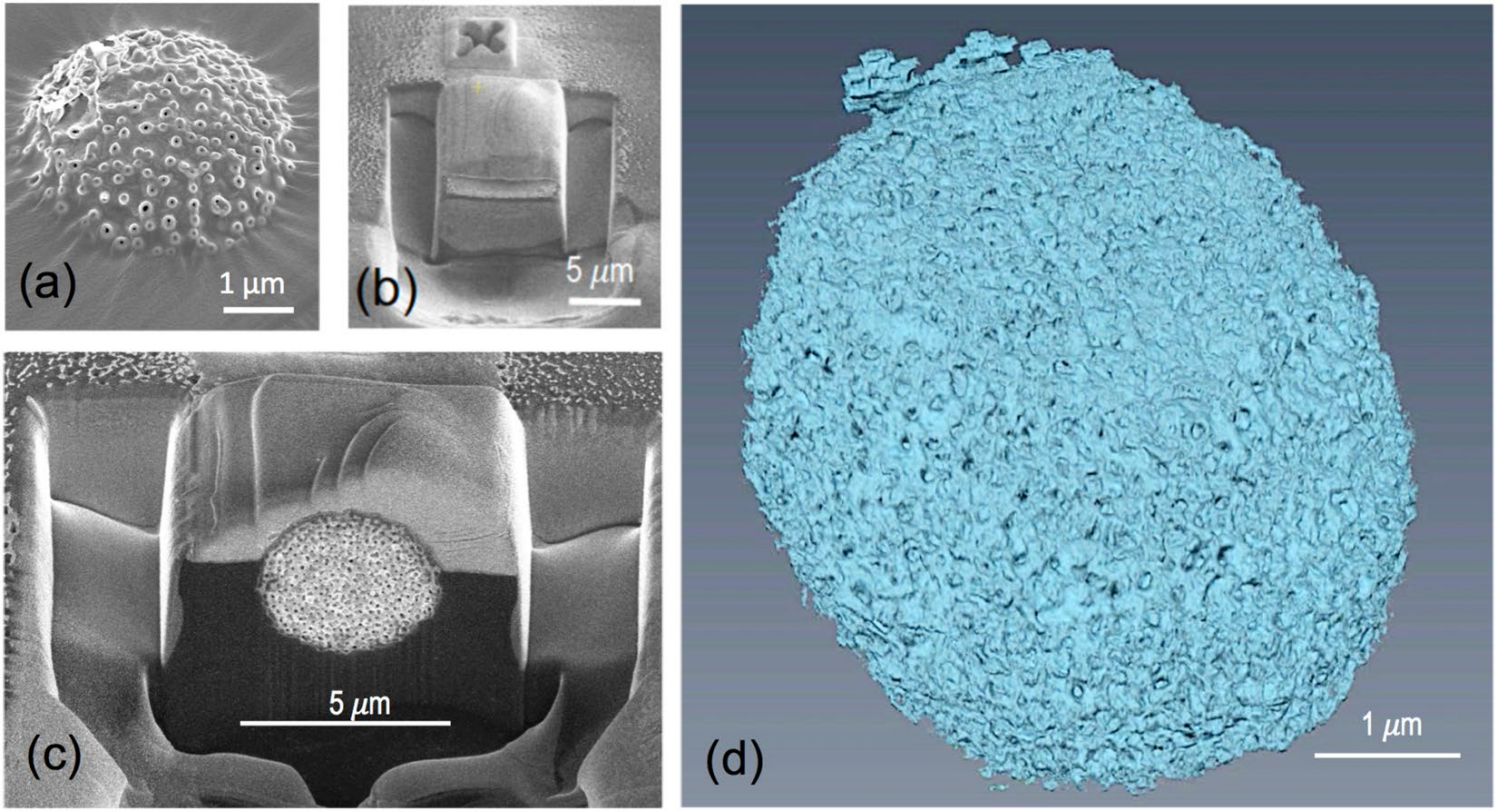
Slice-and-view imaging. (**a**) PS-*b*-PAA porous particles fixed on an adhesive tape; (**b**) covered with a 2.5 μm platinum layer to protect from the ion beam during the trench formation; (**c**) FIB slice-and-view image and corresponding (**d**) 3D reconstruction.

For the particle characterization in this work, slice-and-view FIB/SEM and SBF/SEM were the chosen method, which have a resolution between the TEM and X-ray tomography. FIB/SEM is more commonly applied to inorganic materials, for example zeolites^38^. The application to polymeric materials is more challenging and less explored. Polymeric samples are more sensitive to the ion-beam and the slicing conditions have to be more carefully optimized. The potential for this characterization is high and the application to polymers is now expanding. Examples are the 3D imaging of porous membranes^30^ and hierarchical systems^13^ and chiral gyroid block copolymers^39^ and molecular imprinted polymers^40^. For the FIB/SEM, the samples can be embedded in an epoxy resin, as for TEM, and trimmed for the *in situ* slicing process promoted by the ion beam. For the particles investigated here, this methodology did not provide enough contrast, even after staining. We used another procedure, in which the particles were supported on adhesive tapes, in an analogous way to the method in forensic science to identify shot-gun residues^41^. Figure 3a shows a particle embedded in the adhesive tape.

Figure 3b depicts the particle after a metallic protective coating was deposited on the top of the particle. The gallium ions used during the sectioning have high energies, which can damage the sample, especially in the case of soft materials like polymers, both the carbon and platinum protective layers were deposited before trenching. There were difficulties on sectioning the particle. First, since the glue of the tape was not removed, as it can be seen on the trenches from the SEM images (Fig. 3c), the samples are not perfect cubes, which are typical for harder materials. The solution for this problem was to increase the current of the beam and the time of exposure; the increase of time of operation increase the charging effects over the sample even though the sample was properly sputter-coated with iridium to increase its conductivity. The consequence of the charging in the sample was a constant drifting in the image, which had to be manually corrected, without taking advantage of the automatic tools for the milling and image acquisition. After the manual corrections, a successful series of slices-and-view images was obtained and the resulting 3D reconstruction can be seen in Fig. 3d. A highly porous structure was visualized in the surface and in the bulk of the particles.

While the porosity is clearly seen, the order is not as evident as imaged by SBF/SEM, as shown below. We cannot completely discard that the ion beam could slightly affect the particle fine morphology. The samples for FIB, the optimized sample preparation included a drop deposition and drying step on the silicon wafer, which was done without freezing. For fine porous structures, this could again slightly affect the morphology, but the exposure to RuO_4_ was expected to increase the stability.

Serial Block Face (SBF/SEM) is at least as powerful for porous materials characterization as FIB slice-and-view, with similar high resolution. SBF/SEM can investigate larger volumes of samples than FIB/SEM and could provide a better overview of a collection of particles. SBF/SEM is now been widely used for biological samples^42,43^, but it is not widespread in the characterization of synthetic polymeric materials. We have pioneered the use of SBF/SEM for synthetic membranes^30^ and 3D porous hierarchical polymeric materials^13^. As far as we know SBF/SEM has not been applied to polymer systems and in particular not for porous systems similar to those described here.

**Figure 4:**
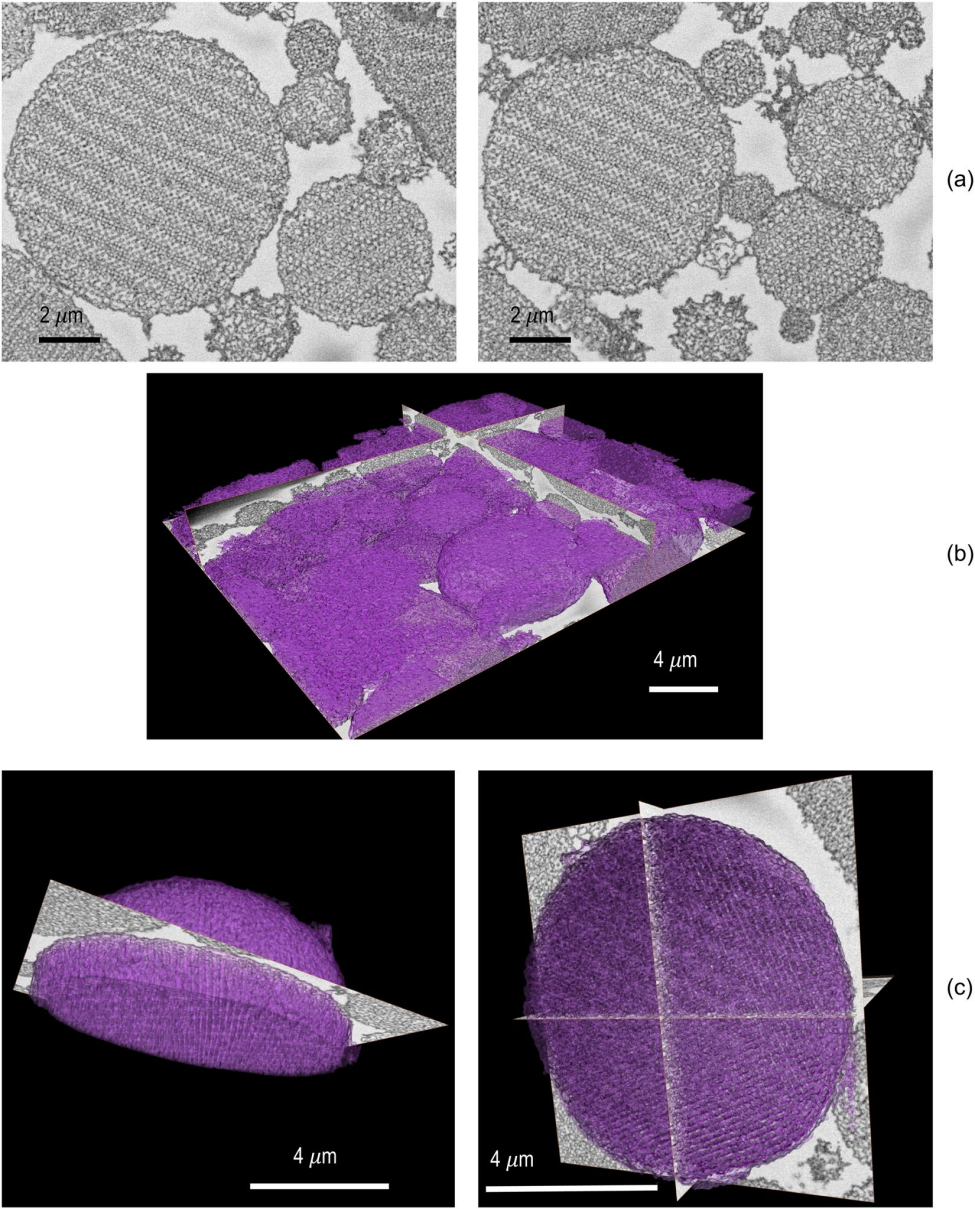
Serial block face (SBF/SEM) images of PS-*b*-PAA porous particles: (**a**) single consecutive sliced images, and (**b**,**c**) 3D reconstructions of a (**b**) collection of particles and a (**c**) single one.

For the SBF/SEM imaging, the volume scope-directional backscattering detector integrated in the Teneo microscope enables the use of epoxy resin blocks containing the particles. Figure 4 shows the images obtained with the SBF/SEM technique. Figure 4a shows two sequential slices of particles previously stained with RuO_4_. It is possible to observe with much more detail the inner structure of the particles with ordered arrays of pores. The microtome inside the microscope mechanically removes layer by layer the slices of the block. In this way there is no sample damage due to the beam, avoiding charging and drifting of the image; thanks to the RuO_4_ staining, the contrast between the resin and the particle was increased for the backscattered electrons, offering higher resolution. The strong ion beam used on FIB might damage the soft sample, but it allowed thinner sections (32 nm) which is convenient to visualize the pores with size around 50 nm. The SBF/SEM imaging was performed with slice thicknesses of 50 and 100 nm, which compromise a fine pore resolution in one dimension of the 3D image reconstruction. But each slice has itself a high horizontal resolution. As a whole the SBF/SEM provided an image (Fig. 4a) with more evident morphological order. Finally, 3D image reconstructions (Fig. 4b,c) were obtained with high resolution.

### SAXS characterization

**Figure 5:**
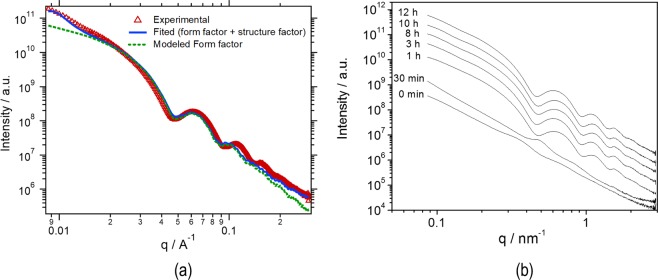
SAXS profile of PS-*b*-PAA porous particles: (**a**) red triangles represent the experimental data and the blue curve is the simulated profile obtained using the cylinder form factor (dashed green curve) and a diffraction peak with maxima at q = 0.009 Å^−1^; (**b**) profiles in different times of evolution.

Even with the predominant ordered cubic structures (Gyroid, Schwarz P) observed by SEM, there is a polydispersity of the particle size and structure as observed in Fig. 1. We assume this is due to the heterogeneity of the process: once the nucleation process initiates, there is a progressive decrease of the copolymer concentration in solution that induces a different kinetics for the next formed nuclei. While the electron microscopy supplied a detailed 3D image of selected single particles, a more statistic information of the overall particles collection can be provided by SAXS. The solution with incipient particles was dried for the measurements. Since the measured q-range corresponds to distances between 2 and 72 nm and the particles are micron size, the morphology probed by SAXS reflects their inner structure. The time evolution of the SAXS curves indicates that this inner structure becomes more regular in particle development times between 30 min and 1 h. From one hour up, the scattering profile in Fig. 5 presents oscillations with deep minima at high *q*, indicative of the structure regularity, while the drop of the intensity at low *q* corresponds to spatial correlations and gives the inter-domain distance of the inner particles structure.

To properly extract the information from the SAXS data, the curve was fitted with a function that comprise a form factor and structure factor. Thus, *I(q)* = S(q)*P(q), where *P(q)* is the form factor and gives information about size and shape of internal structure and the structure factor *S(q)* and gives information about the spatial arrangement of particles. The fitting was made using Irena codes provided with the Igor Pro 6.37 software^44^ and the best approximation was obtained with cylinder as form factor and when a Lorentz diffraction peak was used as structure factor, as illustrated in Fig. 5. The extracted parameters indicated cylinders with about 71 nm length and 8.1 nm mean radius and d-spacing of 69.05 nm between the polymeric phases, assuming cylindrical shapes for modelling the inner structure of the particles. Considering the domains size form in the TEM image (Fig. 2a), the continuous inner channels between the polymer phases have an overall 52.85 nm average diameter. As seen in Fig. 5b, the scattering patterns become more evident as time evolves, indicating that the particles morphology and internal order develops to the final structures depicted by microscopy in Figs 1 to 4. The deepening of the oscillations at high *q* indicates a narrower form factor with increasing the development time, and thus a more monodisperse and regular internal structure.

**Figure 6:**
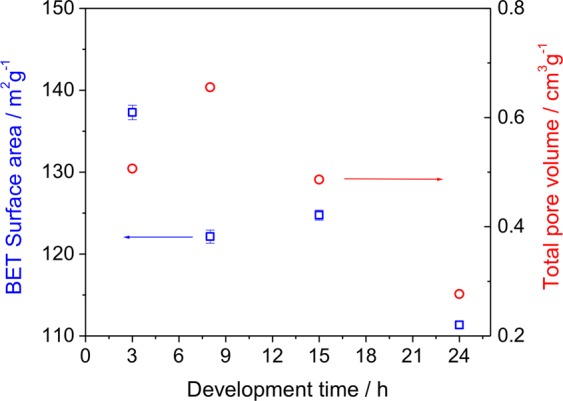
The Brunauer–Emmett–Teller (BET) surface area and total pore volume from N_2_ absorption isotherms for particles with different development time; blue square symbols correspond to the surface area (left axis) and red round symbols were used for the total volume (right axis).

From SEM images, we see in Fig. 1 that not all particles are equally developed, even after 8 hours. Figure 6 shows how the surface area and the total pore volume of the particles change with their developing time. After 15 hours both values decrease, indicating that longer stirring should not be used. A stirring period of 8 hours leads to maximum pore volume, but minimum surface area. We used then particles obtained after 15 hours stirring for the protein sorption/desorption experiments and for the images shown in all Figures shown in this paper.

### Sorption/desorption experiments

The experiments for adsorption and desorption were first carried on with 0.5 mg/mL lysozyme and 0.5 mg/mL porous particles under 3 different pH values (4.4, 5.5 and 6.8). Previous measurements of the ζ-potential^26^ showed that PS-*b*-PAA particles are negative under the investigated pH range. The isoelectric points of lysozyme (molecular weight of 14.3 kg/mol) is 10.7. Therefore, the protein will have positive charges at the whole range of experimental conditions investigated here.

Table 1 shows the results of lysozyme adsorption and desorption at different pH values and two particle concentrations. The absorption values in the table with corresponding errors are averages of 3 to 5 experiments and were calculated based on the lysozyme concentrations in solution, before and after adsorption on the particles, estimated from its UV absorption values in solution. The errors represent standard deviations from the multiple measurements. The desorption was calculated also from UV-absorption in solution, after centrifuging the protein-adsorbed particles and re-dispersing them with NaCl 1M and re-adjusting the pH. As the pH decreases, the amount of lysozyme adsorbed per mg of particle increases, for a fixed particle concentration. At lower pH, the particle ζ-potential becomes less negative, but the particle pores become more open, giving the lysozyme a higher accessibility to the total porosity. The negatively charged carboxylic groups trap the positively charged proteins at low pH. The experiments performed with the particle/protein concentration ratio 0.5:0.5 mg/mL demonstrated the influence of the pH on the adsorption capability. To evaluate if the particle saturation had been achieved under the measured conditions, the protein concentration was increased. When the particle/protein ratio in the solution was changed to 0.05:0.5 for the same fixed volume, the amount of adsorbed protein per mg of particle increased from 0.751 to 1.13 mg. This demonstrated that the particle maximum protein adsorption capacity was even larger and the saturation had not been achieved with the 0.5:0.5 ratio.

**Table 1:** Lysozyme adsorption/desorption experiments.

Particle concentration in adsorption experiments (mg/mL)	pH	Lysozyme (mg) adsorbed per mg of particles	Lysozyme (mg) recovered after desorption per mg of particle	Recovery/ adsorption
0.5	6.8	0.36 ± 0.02	0.077	0.216
	5.5	0. 41 ± 0.10	0.11 ± 0.02	0.270
	4.4	0.73 ± 0.02	0.26 ± 0.04	0.352
0.05	4.4	1.13		

Lysozyme initial concentration in solution: 0.5 mg/mL; particle concentration in desorption experiments: 0.5 mg/mL.

The lysozyme was recovered after desorption, as indicated in Table 1. Higher adsorption naturally leads to a final higher recovery relative to the initial protein amount in the solution. The desorption depends on the ionic strength of the eluent solution, the higher the ionic strength, the higher the competition between the protein and the salt for the ionic bond. The lysozyme desorption efficiency increases with decreasing pH. The pKa of acrylic acid is 4.25. Above this pH, the acid groups are well dissociated, and negative. The electrostatic interactions between the protonated positively charged proteins and the negative particle surfaces favors a strong binding even under the ionic strength provided by 1M NaCl. On the other hand, a pH 4.4 is not far from the acrylic acid pKa value. Therefore, less dissociation is expected, and the particle surfaces are much closer to the neutrality. At pH 4.4, just a small increase of ionic strength is enough to weaken the electrostatic interaction, which governs the protein interaction with the particle. As a consequence, at pH 4.4 the proteins are more easily desorbed than at higher pH.

The particles have therefore interesting gating properties, responding to pH, as confirmed here for lysozyme adsorption/desorption and in previous experiments with immunoglobulin G^26^. The reasons could be a combination of electrostatic effect and pore size. Above pH 4.25, the PAA carboxylic acid groups of the PS_15k_-*b*-PAA_1.6k_ particles are more negative and tend to repel each other, potentially promoting some reduction of the pore sizes. However, compared to pH-responsive PS_138k_-*b*-P4VP_41k_ block copolymer membranes previously reported by our group^45^, the pH effect on the pore size should be minor, since molecular weight of the PAA blocks is only 1600 g/mol, 25-fold shorter than the P4VP blocks in the pore walls of the PS_138k_-*b*-P4VP_41k_ membranes^45^. Before the SEM imaging in this work, the particles were preconditioned above pH 4.25, and freeze-dried. The imaged pore sizes were between 32 and 54 nm, large enough to allow the free sorption of lysozyme (14.3 kg/mol) into the pores.

In summary, different electron microscopy methods for 3D image reconstruction were applied to investigate the ordered triply periodic minimal surfaces morphology of PS-*b*-PAA particles with high sorption capability. While the TEM tomography shows details of a particle in the nanometer scale, two emerging SEM methods provided a more comprehensive image of the porous systems. FIB has been used for ceramics and SBF has a growing application for biological systems. SBF has been used even less for polymer systems than FIB and we demonstrate here the great potential of this methodology.

## Methods

### Materials

Polystyrene-*b*-poly (acrylic acid) (PS-*b*-PAA) copolymer (PS_15k_-*b*-PAA_1.6k_, Mn = 16,600 g mol^−1^, PDI = 1.10) was purchased from Polymer Source, Inc., Canada. Toluene and methanol were supplied by Fischer Scientific. Disodium hydrogen phosphate dihydrate (Na_2_HPO_4_) and potassium dihydrogen phosphate (KH_2_PO_4_) from Sigma Aldrich were used to adjust the pH. Hydrated ruthenium dioxide and sodium periodate (NaIO_4_) were supplied by Sigma Aldrich. Phosphate buffered saline (PBS) solutions were obtained from Thermo Fisher Scientific.

### Particle preparation

The microparticles were prepared by adapting a procedure reported by Yu *et al*.^26^. The best conditions were achieved by dispersing 25.5 mg of PS_15k_-*b*-PAA_1.6k_ in 0.3 mL of toluene and 0.9 mL of methanol (1:3 v/v toluene/methanol), followed by stirring at 1,000 r.p.m for 15 hours. After that, 4.5 mL of methanol were added to 1.2 mL of the initial volume. For the slice-and-view characterization, samples were collected at this stage. For any other experiment, the quenched system was centrifuged at 13,000 r.p.m for 20 min. The supernatant was discarded and the sediments were frozen in liquid nitrogen for 2 hours. Vacuum was then applied overnight under liquid nitrogen, until completely drying.

To define the chosen optimized conditions^26^, the copolymer concentrations were first varied from 0.1 to 4.0 wt% and the volume ratio of methanol to toluene was changed from 0.3:1 to 1:1for the particles preparation. The SEM observation of the collected incipient particles gave the indication of the number of hours requested for the completeness of the formation process. After 8 h stirring, no substantial change was observed anymore, when working with only 10 mg copolymer. However, for batches of 25 mg particles, longer stirring time (15 h) was adopted, since the particles morphology observed by SEM was still progressing after 8 h.

### Protein adsorption/desorption experiments

Lysozyme was dissolved in a low ionic strength (0.01M) PBS buffer solution made with sodium hydrogen phosphate (Na_2_HPO_4_) and potassium dihydrogen phosphate (KH_2_PO_4_) to obtain at pH values of 4.4, 5.5 and 6.8. The concentration of the protein in solution was kept constant at 0.5 mg/mL. The freeze-dried particles were dispersed in 20 mL of the prepared 0.5 mg/mL protein solutions, so that the final particles concentrations were 0.05 and 0.5 mg/mL. The dispersion was magnetically stirred up to 3 days at 200 rpm. The mixtures were centrifuged at 13,000 r.p.m for 20 minutes, and a 5 μL sample droplet of the supernatant was then collected and analyzed using a NanoDrop 2000/2000c Spectrophotometer. The amount of protein adsorbed into the particles was estimated as the product of the volume (20 ml) and the difference in concentration before and after the addition of particles, measured by spectrometry. The procedure was repeated after intervals of 24 hours to confirm that the equilibrium had been reached. For the desorption tests, once the equilibrium was reached and the concentration was measured, the supernatant after the centrifugation was discarded, the remaining particles were immediately resuspended in NaCl solution (1 M), adjusting a particle concentration of 0.5 mg/mL and the pH. The dispersion was stirred at 100 r.p.m for 2 days, and the concentration of the supernatant was measured again by spectrometry to obtain the amount of protein released during the process.

### Morphological characterization

*Staining procedure*. The RuO_4_ solution was prepared adapting a procedure reported by Trent^46^. 0.15 g of hydrated ruthenium dioxide was oxidized by mixing with 25 mL of a 12.8 mg mL^−1^ NaIO_4_ aqueous solution at 1 °C. The samples to be imaged were exposed to the RuO_4_ vapor in a closed system. The RuO_4_ solution was kept at −20 °C for further use.

#### Scanning electron microscopy (SEM)

Unstained freeze-dried particles were collected on aluminum stubs and sputter-coated with platinum or iridium (target supplied by Ted Pella). The secondary electron images were obtained on FEI Nova Nano SEM and Quanta 600 microscopes operating at 2–5 kV.

#### Transmission electron microscopy (TEM) tomography

The freeze-dried particles were stained by exposure to RuO_4_ vapor for 2 hours, following the procedure described above. The particles were then embedded in epoxy resin and cured at 60 °C for 24 h. Slices of 100 nm or thinner were cut with diamond knife using an ultramicrotome Leica EM UC6 and collected on 200–300 mesh copper grids. The TEM tomography was conducted on a FEI Titan CT microscope, operating at 300 kV, equipped with a 4 k × 4 k CCD camera (Gatan, Pleasanton, CA, USA)^47^. The sample was tilted from −65 to +65° and the images were taken at 2° intervals, following a Saxton scheme. The software used for the tomography image acquisition was Xplore 3D. The tomograms were generated using a back-projection algorithm of the IMOD software and the 3D rendering was conducted with Avizo segmentation tools.

#### Sliced-and-view. focused ion beam (FIB)/SEM

30 μL of the particle dispersion (before centrifugation) were drop on a clean silicon wafer. The solution was left to dry at room temperature overnight. The dried particles were stained with ruthenium tetroxide (RuO_4_), by vapor exposure for 2 hours to reduce charging, increase rigidity and provide contrast on the microscope. The staining facilitated the localization of selected particles before initiating the slice-and-view process The stained particles were transferred to an adhesive Scotch^®^ Magic tape by gently pressing it on the silicon wafer, adapting a procedure reported by Niewoehner and Wenz^41^. To prevent charging, the tape was sputter-coated with a 4 nm layer of iridium or platinum. The sample areas containing particles to be sliced and imaged could be easily identified using a Through Lens Detector (TLD), due to RuO_4_ contrast enhancement.

The particles were fully covered with 0.3 nm of carbon, followed by 2.5 μm of platinum coatings to minimize particle damage by the ion beam. Slices of 32 nm thickness were sequentially obtained and imaged on a Helios G4 UX system (Thermo Fisher Scientific) operating with a focused 30 keV beam of gallium ions at 0.36 nA current. The images were acquired with a voltage of 3 kV using a TLD detector for secondary electrons.

#### Serial block face (SBF)/SEM

To determine the internal and external 3D architecture of the block copolymer particles, the samples were analyzed on a Teneo Volume Scope SEM (Thermo Fisher Scientific) equipped with SBF/SEM imaging capability. This consists of an automated *in situ* ultamicrotome for block face sectioning and a Volume Scope-directional backscattering detector for block face imaging. The freeze-dried particles were stained with RuO_4_ for 2 hours to increase the backscattering electrons signal. They were embedded in an epoxy resin and cured in a convection oven at 70 °C overnight. The resin blocks of ca. 3 mm diameter and 0.6 mm thickness, containing the particles, were cut off with jewelry saw and fixed to an aluminum stub with epoxy glue. The block was trimmed with an ultramicrotome (Leica Microsystems) into a cubic shape with the dimensions of 0.5 × 0.5 × 0.4 mm. To reduce charging during imaging, the lateral sides of the block face were coated with colloidal silver glue and the block face was sputter-coated with 10 nm platinum/palladium. Two sets of serial block face images were acquired in an automated mode and in low vacuum mode under the following conditions: accelerating voltage 2 and 2.5 kV, beam current 100 pico Amp, resolution 2048 × 1768, pixel size 9.8 and 12.2 nanometer, slice thickness 50 and 100 nm, chamber pressure 40 Pa.

#### Small-angle X-ray scattering (SAXS)

SAXS measurements were performed at the SAXS1 beamline of the Brazilian Synchrotron facility (LNLS). The beam energy was 8 keV, the X-ray wavelength was 0.155 nm and the sample to detector distance was 1.5 m. For the dry particle preparation, the particles were quenched from the developing solutions by adding a large amount of methanol, followed by a centrifugation and supernatant removal, then drying the sedimentary particles at room temperature for several days. The dry particles agglomerates, resulting from each developing time, were used for the SAXS measurement. An exposure time of 200 s was used and the 2D scattering patterns were recorded by a Pillatus 300k detector with a pixel size of (172 μm)2. The scattering profiles were obtained by radially integration of 2D patterns, after normalization to the intensity of primary beam and subtraction of air scattering. The data was fitted using Igor Pro 6.37 software using a function formed by cylinder form factor and a structure factor represented by a Lorentz diffraction peak.

#### BET measurement

Nitrogen adsorption/desorption experiments isotherms were measured at 25 °C using a Micrometrics ASAP 2420 automated volumetric equipment. To remove the adsorbed materials on the particle surface, the materials were first degassed for 24 h at 25 °C under high vacuum (1.3 × 10^−5^ mbar). The specific surface areas of porous materials were evaluated by the Brunauer−Emmett−Teller (BET) method from the adsorption data in 0.16 to 0.33 relative pressure range. The total pore volume was estimated from a single point adsorption at a relative pressure of 0.996.
